# Structural Characterization of Human Bufavirus 1: Receptor Binding and Endosomal pH-Induced Changes

**DOI:** 10.3390/v16081258

**Published:** 2024-08-06

**Authors:** Mitchell Gulkis, Mengxiao Luo, Paul Chipman, Mario Mietzsch, Maria Söderlund-Venermo, Antonette Bennett, Robert McKenna

**Affiliations:** 1 Department of Biochemistry and Molecular Biology, University of Florida, Gainesville, FL 32611, USA; mitchell.gulkis@ufl.edu (M.G.); mxluo@connect.hku.hk (M.L.); pchipman@ufl.edu (P.C.); mario.mietzsch@ufl.edu (M.M.); 2 Department of Virology, University of Helsinki, P.O. Box 21 (Haartmaninkatu 3), FIN-00014 Helsinki, Finland; maria.soderlund-venermo@helsinki.fi

**Keywords:** parvoviruses, bufavirus, single-stranded DNA virus, cryo-EM and image reconstruction

## Abstract

Bufaviruses (BuV) are members of the *Parvoviridae* of the *Protoparvovirus* genus. They are non-enveloped, T = 1 icosahedral ssDNA viruses isolated from patients exhibiting acute diarrhea. The lack of treatment options and a limited understanding of their disease mechanisms require studying these viruses on a molecular and structural level. In the present study, we utilize glycan arrays and cell binding assays to demonstrate that BuV1 capsid binds terminal sialic acid (SIA) glycans. Furthermore, using cryo-electron microscopy (cryo-EM), SIA is shown to bind on the 2/5-fold wall of the capsid surface. Interestingly, the capsid residues stabilizing SIA binding are conserved in all human BuVs identified to date. Additionally, biophysical assays illustrate BuV1 capsid stabilization during endo–lysosomal (pH 7.4–pH 4) trafficking and capsid destabilization at pH 3 and less, which correspond to the pH of the stomach. Hence, we determined the cryo-EM structures of BuV1 capsids at pH 7.4, 4.0, and 2.6 to 2.8 Å, 3.2 Å, and 2.7 Å, respectively. These structures reveal capsid structural rearrangements during endo–lysosomal escape and provide a potential mechanism for this process. The structural insights gained from this study will add to the general knowledge of human pathogenic parvoviruses. Furthermore, the identification of the conserved SIA receptor binding site among BuVs provides a possible targetable surface-accessible pocket for the design of small molecules to be developed as anti-virals for these viruses.

## 1. Introduction

Human bufavirus 1 (BuV1) was initially identified in stool samples of patients suffering from acute gastroenteritis in Burkina Faso, hence the name bufavirus [1]. Since its discovery, many other epidemiological reports have detected BuV1 in patients spanning five continents, likely indicating worldwide distribution [1,2,3,4,5,6,7,8,9,10,11,12,13,14,15,16]. Strikingly, one study reported that antibodies against BuV1 are prevalent in Iraq (84.8%), Kenya (72.3%), and Iran (56.1%), while being less common in the United States (3.6%) and Finland (1.9%) [2]. BuV1 has been found mostly in diarrheal but also rarely in non-diarrheal stool samples, suggesting a putative causative link between BuV1 and gastroenteritis. Additionally, two other viruses have been discovered, which were classified as bufavirus genotypes through homology to the BuV1 non-structural proteins and named human bufavirus 2 and 3 (BuV2 and BuV3) [1,10]. To date, these viruses have shown no cross-reactivity, indicating that they are distinct serotypes [11]. Bufaviruses have also been found in many animals, including dogs, cats, pigs, bats, rats, and shrews, demonstrating that they are not restricted to human hosts [17,18,19,20,21,22].

BuV1 is a member of the *Protoparvovirus* genus of the *Parvoviridae* family. Parvoviruses are non-enveloped ssDNA viruses with a T = 1 icosahedral capsid of approximately 25 nm in diameter [23,24,25,26,27,28]. Other members of this genus include the minute virus of mice (MVM), feline parvovirus (FPV), canine parvovirus (CPV), tusavirus (TuV), cutavirus (CuV), and rat H-1 parvovirus (H-1PV), most of which are pathogenic to their respective hosts [23,27,29,30]. Previous studies have shown that minor differences on the exterior of the viral capsid can have major impacts on their pathogenicity, perhaps best exemplified by the differences between MVMi and MVMp, where twelve amino acid differences affect capsid binding to cell surfaces and determine pathogenicity and tropism [31,32,33,34]. Therefore, it is important to determine how the capsids of these parvoviruses are structured during various steps in their viral lifecycle to better understand their pathogenicity. 

The BuV1 capsid packages a ssDNA genome of approximately 5000 nt in length. The genome encodes three open reading frames (ORFs): the NS ORF encodes non-structural (NS) proteins used during viral replication and packaging, the VP ORF encodes the structural viral proteins (VPs), which assemble into the capsid, and the third ORF is of unknown function [1,24,25,35]. From the *VP* ORF, two overlapping proteins are translated, VP1 and VP2, which are incorporated into the viral capsid in a ~1:10 ratio. VP1 contains a unique region at its N-terminus, referred to as VP1u, which is predicted to encode a phospholipase A2 (PLA_2_) domain. PLA_2_ activity is required for endo–lysosomal escape after receptor-mediated endocytosis [23,24,25,26,27]. The structures of several parvoviruses have been determined by X-ray crystallography and cryo-electron microscopy (cryo-EM) [23]. To date, there is no available structure for VP1u. Similar to all known parvoviruses, BuV1 assembles into a T = 1 icosahedral capsid. The gross capsid morphology includes pores at the 5-fold vertices, protrusions surrounding the 3-fold axes, a depression region at the 2-fold axes, and a wall separating the depressed regions near the 2- and 5-fold axes [36]. The VPs have an eight-stranded β-barrel motif and an α-helix that form the core of the capsid, which is characteristic of all members of the *Parvoviridae* [36]. Between strands in the β-barrel, large loops extend outward to form the unique surface features of the viral capsid [23,36]. These loops create structural differences between viruses, which have been previously categorized into variable regions (VRs) [23,37]. 

To infect host cells, protoparvoviruses, like most viruses, must undergo a multi-step trafficking pathway before viral replication can be initiated [23,26,27]. First, the virus must attach to a host cell and trigger receptor-mediated endocytosis. Previous studies have shown that this is commonly mediated by surface-exposed glycans such as sialic acid (SIA), heparan sulfate proteoglycan (HSP), or galactose (GAL) [27,38]. Other mechanisms of cell attachment have also been demonstrated, including binding to proteinaceous receptors [27]. Following receptor-mediated endocytosis, protoparvoviruses enter the early endosome and begin trafficking through the endo–lysosomal pathway. The maturation of early endosomes to late endosomes and, finally, lysosomes results in continuous acidification of the endo–lysosomal compartment [27]. This change in pH has been previously suggested to cause structural rearrangements of protoparvovirus capsids, resulting in the externalization of VP1u, which allows the PLA_2_ domain to degrade the endo–lysosomal membrane, mediating viral escape into the cytoplasm [23,26,27,39,40]. Once in the cytoplasm, parvoviruses traffic towards the nucleus, across the nuclear membrane, and eject their genome, where host factors recognize and replicate the viral genome [26,27].

Despite the widespread distribution and prevalence of BuV1, much is unknown about its viral lifecycle, especially at the molecular level. Here, the capsid is biophysically studied, and its structure is determined in conditions mimicking intermediates along its putative viral lifecycle. Additionally, SIA is identified as the terminal glycan receptor for BuV1, and its capsid binding pocket is identified on the 2/5-fold wall. This site appears to be conserved in pathogenic protoparvoviruses and represents a possible druggable site to prevent viral infection. Furthermore, the cryo-EM structures of BuV1 at pH 7.4, 4.0, and 2.6 highlight structural rearrangements of the capsid, demonstrating capsid stability at different pHs and providing insights into the pH-induced widening/narrowing of the 5-fold channel to facilitate VP1u externalization and endo–lysosomal escape. 

## 2. Materials and Methods

### 2.1. Expression and Purification of Bufavirus 1 Virus-like Particles

The portion of the BuV1 capsid gene encoding VP2 was cloned into the pFastBac1 plasmid and used to generate recombinant baculoviruses using the Bac-to-Bac procedure as previously described [36]. Sf9 insect cells were cultured in Grace’s medium (Invitrogen, Waltham, MA, USA) with 10% fetal bovine serum and 1% antibiotic/antimycotic (Gibco, Waltham, MA, USA). The cells were infected with the recombinant baculovirus at an MOI of 5 and harvested after 72 h. The cell pellet was harvested by centrifugation at 3000 rpm for 20 min at 4 °C in a Beckman JA-20 rotor (Beckman Coulter, Brea, CA, USA). The pellet was stored at −20 °C in lysis buffer (50 mM Tris HCl pH 8.0, 100 mM NaCl, 1 mM EDTA, and 0.2% (*v*/*v*) Triton X-100) until purification. Cells were lysed by three freeze–thaw cycles of 15 min at 37 °C, followed by 2 min in liquid nitrogen (−196 °C). Following lysis, to digest contaminating DNA and RNA, 1 μL (25 units) of benzonase (Millipore, Burlington, MA, USA) was added for every 10 mL of resuspended cells and incubated at 37 °C for 1 h. The cell lysis was then clarified by centrifugation at 10,000 rpm for 30 min at 4 °C in a Beckman JA-20 rotor (Beckman Coulter). The clarified supernatant was applied to the top of 5 mL of 20% sucrose in TNTM buffer (25 mM Tris HCl pH 8.0, 100 mM NaCl, 0.2% (*v*/*v*) Triton X-100, and 2 mM MgCl_2_) and subjected to ultracentrifugation at 45,000 rpm for 3 h at 4 °C. The resultant pellet was resuspended in 1 mL TNTM and applied on top of a 5%–40% step sucrose gradient in TNTM. The gradient was ultracentrifuged at 35,000 rpm for 3 h at 4 °C using a Beckman SW41-Ti rotor (Beckman Coulter). Visible blue bands were extracted from the gradient at ~20% sucrose concentration using a syringe, and the sample was dialyzed into 1X PBS (2.8 mM KCl, 137 mM NaCl, 10 mM Na_2_PO_4_, and 1.8 mM KH_2_PO_4_) and stored at 4 °C. 

### 2.2. Sample Purity

To determine sample purity and concentration, 10 μL of the sample was loaded on a 10% SDS-PAGE gel with a protein ladder and increasing concentrations of bovine serum albumin (BSA) to determine purity and capsid concentration. The concentration was determined by densitometry using the BSA lanes as standards. 

### 2.3. Fluorescent Labeling of VLPs

The VLPs were fluorescently labeled using the DyLight 488 antibody labeling kit (Thermo Fisher, Waltham, MA, USA) following a modified version of the manufacturer’s protocol. A total of 40 µL of borate buffer (0.67 M, pH 8.5) was added to 500 µL of the VLPs at a concentration >0.5 mg/mL, mixed, and transferred to the DyLight reagent vial. The mixture was incubated for 1 h at RT, protected from light. Unbound fluorescent molecules were removed from the sample by dialysis using a membrane with a 30 kDa cutoff into 4 L of 1× PBS. The dialysis was performed at 4 °C overnight with slow stirring utilizing a magnetic stirrer. The dialysis buffer was changed two additional times after 3 h of dialysis. The success of the labeling procedure was confirmed by SDS-PAGE, which showed fluorescent VP2 bands when viewed under UV light.

### 2.4. Glycan Array

Fluorophore-labeled VLPs were analyzed on a glycan microarray for their glycan-binding ability at the Emory Comprehensive Glycomics Core. The procedure was described previously [41]. In brief, 600 different glycan structures are printed on microscope glass slides (CFG glycan array V5.2), each in replicates of six. The samples at ~180 µg/mL, supplemented with 1% BSA and 0.05% Tween-20, were incubated on the glycan array for 1 h at room temperature in a dark, humidified chamber. The slides were washed in PBS with 0.05% Tween-20, dried by spinning, and scanned by an Innopsys scanner using the 488 nm wavelength laser. The data sets were analyzed by averaging the data for four replicates after the elimination of two spots with the highest and lowest intensity.

### 2.5. Cell Binding Assay

Low passage Chinese hamster ovary (CHO) cell lines Pro5 and Lec2 were cultured as monolayers in MEM-α (ATCC) with 10% FBS (fetal bovine serum) and 1% antibiotic/antimycotic (Gibco) in a 5% CO_2_, 37 °C incubator. For cell binding assays, the CHO cells were detached from plates by addition of EDTA, pelleted, resuspended in MEM-α to 5 × 10^5^ cells/mL, pre-chilled for 30 min at 4 °C, and aliquoted to 500 µL fractions. Each tube of cells was then incubated with the fluorescently labeled VLPs at a MOI of 10^6^ under constant rotation for 3–4 h at 4 °C (protected from light). Following the incubation, the cells were pelleted at 2000 rpm in a Beckman JA-10 rotor for 10 min, and the supernatant was discarded. Unbound VLPs were removed by washing the cells with 300 µL ice-cold 1× PBS, followed by centrifugation. Pellets were resuspended in 300 µL 1× PBS and analyzed utilizing a FACS Canto (BD, Franklin Lakes, NJ, USA). Cells without added fluorescent-labeled capsids were used as a baseline to determine the percentage of fluorescent cells in the other samples. All experiments were conducted in triplicate. The FSC Express5 software suite (De Novo Software, Pasadena, CA, USA) was used to analyze the raw data.

### 2.6. Differential Scanning Fluorimetry

To probe the effect of pH on the thermal stability of the BuV1, 10 μL of capsids at 0.5 mg/mL were mixed with 90 μL of universal buffer (20 mM HEPES, 20 mM MES, 20 mM Sodium Acetate, 150 mM NaCl) adjusted to the pH specified and allowed to equilibrate for 15 min. Aliquots of 22.5 μL of this diluted sample were then mixed with 2.5 μL of 1% (*v*/*v*) SYPRO orange (Life Technologies, Carlsbad, CA, USA) in a 96-well PCR plate. In a PCR block equipped with fluorescent detection using the BioRad CFX Connect device (BioRad, Hercules, CA, USA), the sample was first heated to 30 °C for 10 min, then the temperature increased by 0.5 °C every 6 s until it reached 99 °C. At each temperature, a fluorescent reading was taken. The melting temperature (T_m_) was defined as the peak of the first derivative of fluorescence (dF/dT).

### 2.7. Vitrification and Cryo-Electron Microscopy Data Collection

Purified BuV1 VLPs were dialyzed to pH 2.6 in 50 mM glycine HCl and 150 mM NaCl and to pH 4.0 and pH 7.4 in universal buffer (20 mM HEPES, 20 mM MES, 20 mM sodium acetate, and 150 mM NaCl). Three microliters of purified BuV1 VLPs (~1 mg/mL) at their respective pH conditions were applied to Quantifoil R 2/2 grids with a 2 nm carbon layer (Electron Microscopy Sciences, Hatfield, PA, USA) and vitrified using a Vitrobot™ Mark IV (FEI Co., Waltham, MA, USA). The capsid distribution and ice quality of the grids were examined using a 16-megapixel CCD camera (Gatan, Inc., Pleasanton, CA, USA) in a Tecnai (FEI Co.) G2 F20-TWIN transmission electron microscope operated at a voltage of 200 kV using low dose conditions (~20 e/Å^2^). Optimal grids were used for collecting micrograph movie frames using the Leginon semi-automated application on a Titan Krios electron microscope (FEI Co.) operated at 300 kV with images recorded on a Gatan K2 Summit direct electron detection camera. The microscope was equipped with a Gatan post-column imaging filter (GIF) utilizing a slit width of 20 eV. Data collection used counting mode and an accumulated dose of 75 e^−^/Å^2^ fractionated into 50 movie frames per micrograph. The data sets were collected as part of the National Institutes of Health’s (NIH) “West/Midwest Consortium for High-Resolution Cryo Electron Microscopy” project. A nominal magnification of 130,000× was used for data collection, resulting in a pixel size of ~1.1 Å. The data collection parameters are provided in Table 1.

### 2.8. Data Processing and 3D Reconstruction

Raw movies were aligned using MotionCor2 with dose weighting [42]. Aligned micrographs were then imported and processed using cryoSPARC [43]. Briefly, the contrast transfer function was fitted using the Patch CTF Estimation module of cryoSPARC. The blob picker module was used with a particle diameter of 250 Å to pick initial particles, which were manually curated and then subjected to 2D classification. Two-dimensional classes that resembled the expected icosahedral geometry were used to generate a template. Particles were then repicked using the template picker module with a particle diameter of 250 Å and box size of 500 Å. These particles were manually curated and used for 2D classification before final 2D classes were selected, which most resembled the expected icosahedral geometry. An initial 3D *ab initio* model was generated to a maximum resolution of 12 Å with C1 symmetry imposed. This was further refined using homogenous refinement imposing icosahedral (I) symmetry and sharpened using a linear fit to the Guinier plot to generate the final sharpened electron density map (6SLN: −90.4 Å^2^, pH 7.4: −154.6 Å^2^, pH 4.0: −174.8 Å^2^, and pH 2.6: −166.7 Å^2^). For the BuV1 6SLN data set, it was necessary to generate a less sharpened map (B = −50.4 Å^2^) to determine the location of the bound glycan, and this is indicated in the figure legend.

### 2.9. Model Building and Structure Refinement

The previously determined structure of BuV1 determined at pH 7.4 (PDB: 6BWX) was docked into the final density maps using Chimera [44]. Both pixel size and map handedness were adjusted to maximize the correlation coefficient between the experimental density map and a map generated around the model using the same resolution as the experimental density map. A monomer was then extracted from the complete 60-mer and manually fit using Coot [45]. This monomer was then used to generate a 60-mer using the ViperDB Oligomer Generator [46]. The 60-mer was then refined in PHENIX using real-space refinement [47,48]. Finally, manual adjustments were made to correct Ramachandran and rotamer outliers that did not have a clear density to support them. The refinement statistics reported are from the log of the phenix.refine file [47]. Final models and maps were overlayed in Coot, and structural differences were manually inspected. Figures were made using ChimeraX and PyMOL (The PyMOL Molecular Graphics System, Version 2.5.2, Schrödinger, LLC., New York, NY, USA) [49].

## 3. Results

### 3.1. BuV1 Binds to Sialic Acid

**Figure 1:**
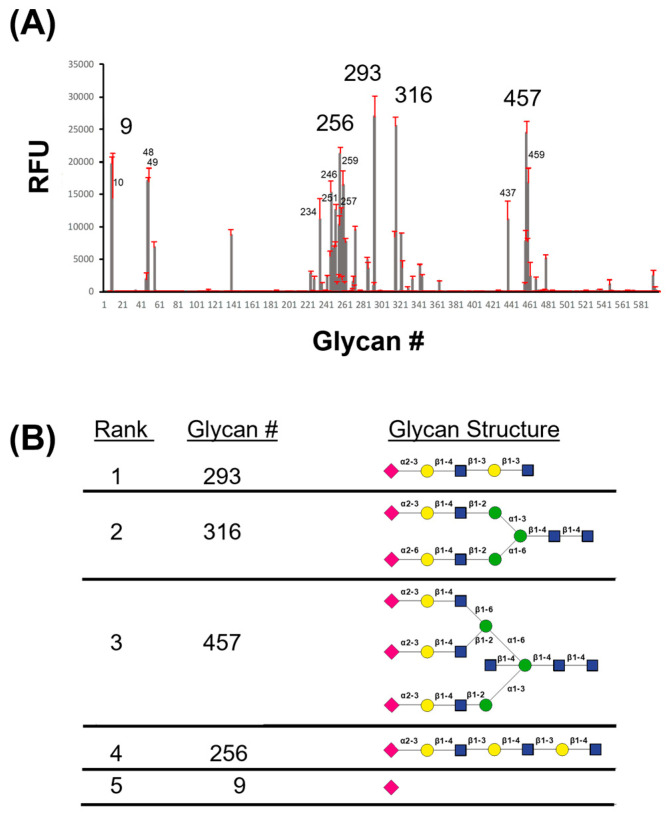
Glycan array of BuV1 demonstrates sialic acid binding. (**A**) Relative fluorescence units for each of the 600 glycans are shown with the standard deviation in red. The numbers of the 15 glycans with the highest signal are displayed, with the top 5 glycans shown in larger text. (**B**) Table showing the symbols of the glycan structure of the top five glycans with the highest signal. Pink diamonds represent N-acetylneuraminic acid, yellow circles represent galactose, blue squares represent N-acetylglucosamine, and green circles represent mannose. Glycosidic linkages are indicated between sugars.

Previous studies have shown that many parvoviruses utilize glycans to promote host cell attachment [23,24,25,26,27,38]. Following confirmation of VLP purity via SDS-PAGE analysis, BuV1 capsids were labeled with DyLight 488 and subjected to a high throughput glycan array to determine their glycan-binding profile (Figure 1). From this analysis, it was observed that the BuV1 capsid preferentially bound to glycans with a terminal SIA with either α2-3 or α2-6 linkage to galactose attached by a β1-4 linkage to N-acetylglucosamine. However, the BuV1 capsid was also observed to bind to SIA alone (glycans #9 and #10), which suggests that the terminal SIA moiety alone may be the determining factor for binding to the cell surface, regardless of the linkage or the presence of galactose as the second molecule in the glycan chain (Figure 1 and Supplementary Figure S1). 

**Figure 2:**
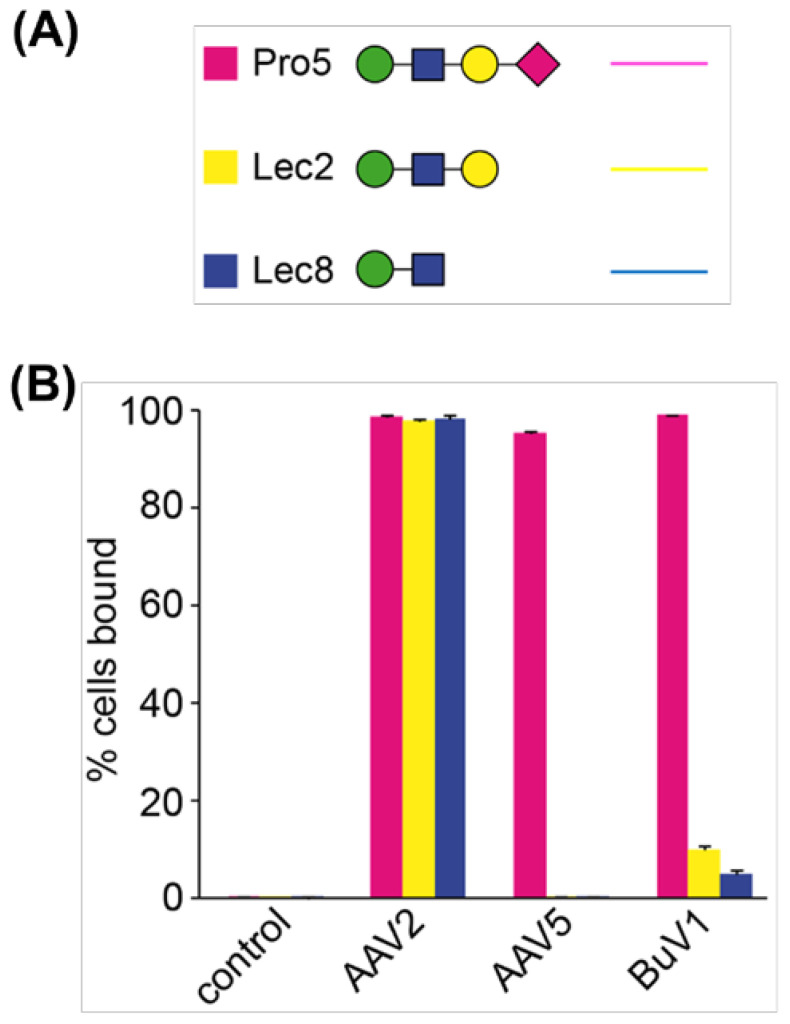
BuV1 utilizes sialic acid for cell adhesion. (**A**) Glycan symbols show the glycans expressed on the surface of Pro5 cells (pink), Lec2 cells (yellow), and Lec8 cells (blue). Pink diamonds represent N-acetylneuraminic acid, yellow circles represent galactose, blue squares represent N-acetylglucosamine, and green circles represent mannose. The rightmost glycan is the terminal glycan. (**B**) Bar graphs show the percentage of fluorescently labeled cells compared to the total cell count. Bars are colored as in (**A**).

In order to determine whether BuV1 uses SIA for host–cell attachment, a FACS-based cell binding assay was used. Here, fluorescently labeled capsids were incubated with CHO cells presenting glycans with a terminal sialic acid (Pro5), a terminal galactose (Lec2), or a terminal N-acetylglucosamine (Lec8). After washing, the cells were analyzed using FACS to determine the fraction of total cells that were fluorescent due to BuV1 binding. AAV2 was used as a positive control since it is known to bind to heparan sulfate proteoglycan, which is expressed in all three CHO cell lines [38,50]. AAV5 has been previously demonstrated to use terminal SIA but did not use either galactose or N-acetylglucosamine to mediate cell adhesion; therefore, it was used as a benchmark [38,51,52]. Interestingly, BuV1 capsids exhibited robust binding to Pro5 cells, with only minimal binding to Lec2 or Lec8 cells, demonstrating that BuV1 uses terminal SIA to mediate cell adhesion (Figure 2). 

### 3.2. BuV1 Binds Sialic Acid at the 2-/5-Fold Wall

**Figure 3:**
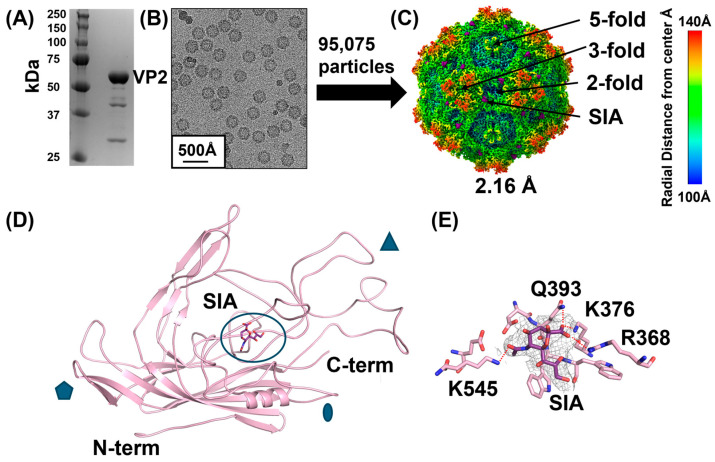
Structural characterization of BuV1 in complex with sialic acid. (**A**) SDS-PAGE gel of purified BuV1 capsids at pH 7.4. (**B**) Representative micrograph of BuV1 VLPs embedded in vitreous ice. A scale bar is given in the lower left corner. (**C**) 2.16 Å reconstructed electron density map shown with a sharpening B factor of −90 at 2σ. The surface is colored radially, as indicated in the scale bar to the right. Locations of bound sialic acid are shown in purple. (**D**) Cartoon structure of a BuV1 monomer is shown in pink. The bound sialic acid is shown in stick form in purple. The monomer is shown with regions participating in icosahedral symmetry elements on the exterior labeled with blue shapes. The 5-fold axis is denoted by the pentagon, the 3-fold by the triangle, and the 2-fold by the ellipse. (**E**) The interacting pocket is shown in detail with residues (pink) labeled, which hydrogen bond with the sialic acid (purple). Electron density is shown for the sialic acid at 1σ with a sharpening B factor of −50 Å^2^.

To identify the SIA binding site on the BuV1 capsid, BuV1 VLPs composed of only VP2 were purified. Additional bands on the SDS-PAGE gel were confirmed as cleavage products of VP2 by mass spectrometry (Figure 3A). BuV1 VLPs were then co-incubated with either NeuNAcα2-3Galβ1-4GlcNAc (3SLN) or NeuNAcα2-6Galβ1-4GlcNAc (6SLN) in 10-fold molar excess of the potential binding sites. Cryo-EM data was collected for both samples (Figure 3B). For the BuV1-6SLN sample, 95,075 particles were extracted, and the 3D reconstruction resulted in a 2.16 Å electron density map (Figure 3C). The previously determined structure of BuV1 (determined at pH 7.4) was docked into this map and then manually refined [36]. The quality of the electron density map allowed for the assignment of most side-chain conformations (Supplementary Figure S2A). However, a surface loop between L495 and N502 exhibited poor density, as previously described, likely due to loop flexibility (Supplementary Figure S2B) [36]. The BuV1-6SLN model, after refinement, was experimentally identical to the previously deposited BuV1 model with a Cα-RMSD of 0.26 Å.

Additional density not attributed to the protein model was clearly visible along the 2-/5-fold wall (Figure 3C–E). Interestingly, only the terminal SIA was able to be fitted into the density in a conformation that leaves the 2′-oxygen exposed to bulk solvent (Figure 3D,E). It is likely that the other two sugar moieties are not visible in the cryo-EM reconstruction due to the flexibility of these moieties in bulk solution, as they are unlikely to interact with the BuV1 capsid, consistent with the observations of the glycan array. This structure showed that the SIA formed five hydrogen bonds with the capsid. The ε-amine of K545 with SIA O10, the backbone carbonyl of Q393 with N5, both the amino nitrogen of Q393 and the ε-amine of K376 to O1B, and the guanidinium moiety of R368 with O1A. Analysis of the binding site by PDBePISA calculated that the sialic acid occludes ~250 Å^2^ of the capsid surface. 

The 3D reconstruction of the BuV1-3SLN data set produced a 2.57 Å density map, also with additional density not attributable to the capsid, in the same location as the fitted SIA in the BuV1-6SLN. This confirms the findings of the BuV1-6SLN data set but also implies that the linkage to galactose is not important for SIA binding to BuV1.

### 3.3. BuV1 Is More Stable at Lower pH

**Figure 4:**
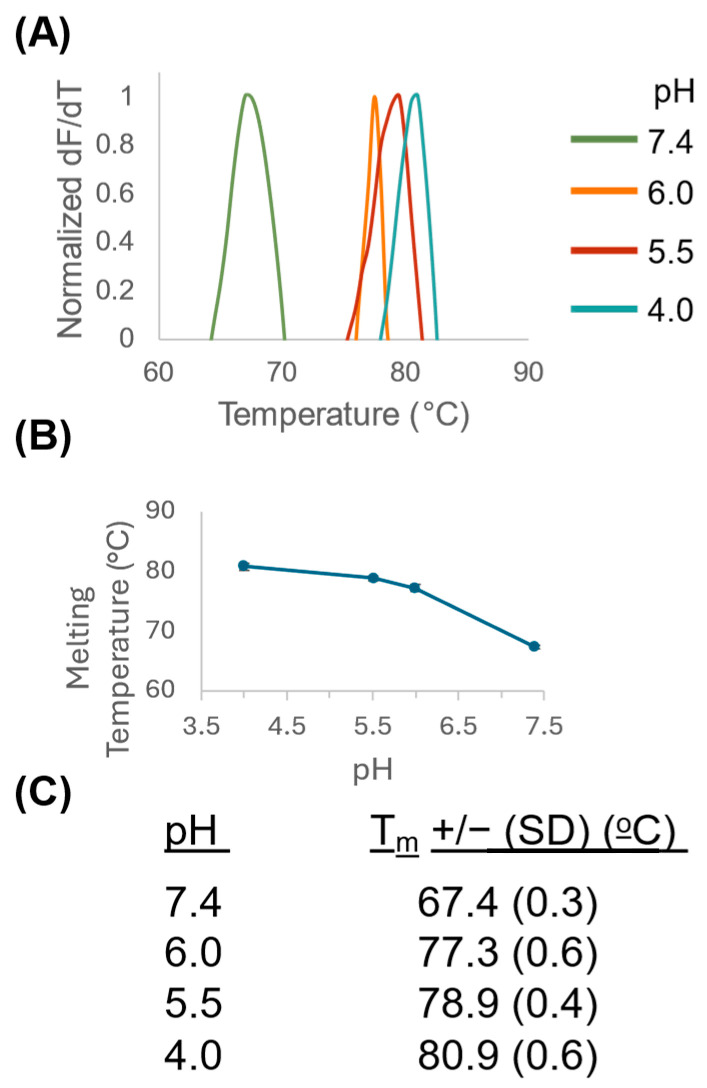
BuV1 shows greater thermal stability at lower pHs. (**A**) Representative traces of the first derivative of measured fluorescence are shown for differential scanning fluorimetry experiments conducted at pH 7.4 (green), pH 6.0 (orange), pH 5.5 (red), and pH 4.0 (teal). (**B**) Graph, which shows melting temperature as a function of pH. (**C**) Table showing the melting temperature (T_m_) of BuV1 at different pHs. Each experiment was performed on three separate days, with three technical repeats on each day.

After receptor-mediated endocytosis, parvoviruses experience continuous acidification in the endo–lysosomal pathway, from pH 7.4 to 6.0 (early endosome), 5.5 (late endosome), and 4.0 (lysosome), which is thought to induce structural rearrangements leading to externalization of VP1u and lysosomal escape [23,26,27,40]. To test the stability of the BuV1 capsid at these physiologically relevant pHs, differential scanning fluorimetry was employed. Analysis of the melting temperature (T_m_) showed a robust increase in the thermal stability of the BuV1 capsid at lower pHs (Figure 4). The T_m_ of 67.4 °C for BuV1 at pH 7.4 is consistent with previously reported values [36]. An approximate increase of 10 °C was observed between pH 7.4 and pH 6.0, while a more modest increase was also observed between pH 6.0 and pH 5.5, as well as between pH 5.5 and pH 4.0 (Figure 4B). Since BuV1 was originally discovered in and is associated with patients with gastrointestinal distress, we attempted to characterize the thermal stability of BuV1 at pH 2.6 to mimic the acidic environment of the stomach [53,54,55]. However, no peaks were detected for the experiment at pH 2.6. The cryo-EM micrographs clearly show both full and half (broken) capsids at this pH; therefore, it is unclear whether the lack of signal is due to the fluorescent dye or if some of the hydrophobic patches of the capsid are exposed before heating. 

### 3.4. Structural Characterization of BuV1 at pH 7.4, 4.0, and 2.6

**Figure 5:**
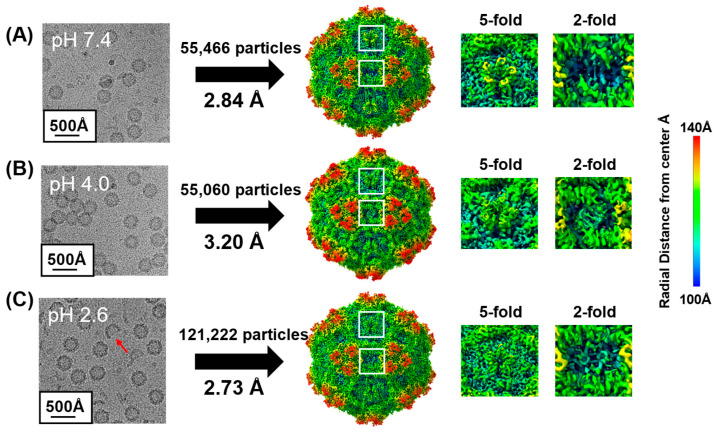
Reconstruction of BuV1 capsids at pH 7.4, 4.0, and 2.6. (**A**–**C**, **left**) Representative micrographs are shown for BuV1 at pH 7.4 (**A**), 4.0 (**B**), and 2.6 (**C**). A scale bar is given in the lower left corner. A partial capsid is indicated with a red arrow (**C**). (**A**–**C**, **middle**) Sharpened reconstructed electron density maps for BuV1 at pH 7.4 (**A**), 4.0 (**B**), and 2.6 (**C**) are shown at 2σ. The surface is colored radially, as indicated in the scale bar to the far right. (**A**–**C**, **right**) Zoomed view of the 5-fold and 2-fold symmetry axes for the reconstructed electron density maps for BuV1 at pH 7.4 (**A**), 4.0 (**B**), and 2.6 (**C**) shown at 2σ. The surface is colored radially, as indicated in the scale bar to the far right.

In order to characterize the structural rearrangements that may occur under various pHs relevant to the viral lifecycle, BuV1 capsids were dialyzed to pH 7.4, 4.0, or 2.6, vitrified on cryo-EM grids, and subjected to cryo-EM data collection (Table 1). Subsequent 3D reconstruction yielded electron density maps at 2.84, 3.20, and 2.73 Å for pH 7.4, 4.0, and 2.6, respectively (Figure 5). The map quality allowed for the determination of the conformations for most of the side chains in the capsid (Supplementary Figure S2B–D). A notable exception is that the surface loop between L495 and N503 in the HI loop was not well defined at pH 7.4 or 4.0 (Supplementary Figure S2F–G). However, reasonable electron density allowed the fitting of this loop at pH 2.6 (Supplementary Figure S2H). Therefore, to prevent overfitting, the coordinates for the HI loop at pH 2.6 were used at both pH 7.4 and pH 4.0. Qualitative inspection of the density maps showed clear differences at both the 5- and 2-fold symmetry axes at lower pHs (Figure 5). At the 5-fold symmetry axis, the pore was visually larger at pH 4.0, compared to both pH 2.6 and pH 7.4 (Figure 5, right). Furthermore, there were clear pH-dependent rearrangements at the 2-fold symmetry axis, suggesting conformational dynamics (Figure 5, right). 

**Figure 6:**
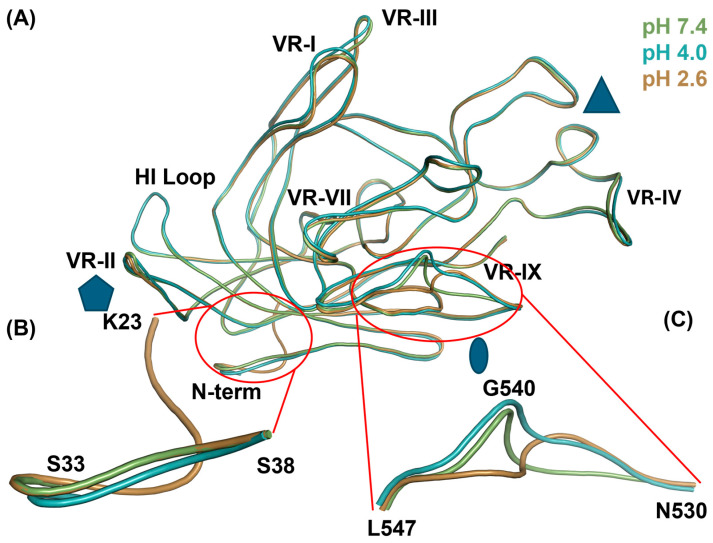
BuV1 monomers at pH 7.4, 4.0, and 2.6 reveals pH-dependent differences. (**A**) Overlay of a monomer from the final 60-mer models of the BuV1 capsid at pH 7.4 (green), 4.0 (teal), and 2.6 (tan). The monomers are aligned using secondary structure matching to the BuV1 pH 7.4 monomer. The monomer is shown with regions participating in icosahedral symmetry elements on the exterior labeled with blue shapes. The 5-fold axis is denoted by the pentagon, the 3-fold by the triangle, and the 2-fold by the ellipse. (**B**) Zoomed view of the differences in the N-terminal observed between models of the BuV1 capsid at pH 7.4 (green), 4.0 (teal), and 2.6 (tan). Ordering of 10 additional residues is observed at pH 2.6 (tan). (**C**) Zoomed view of the differences in VR-IX observed between models of the BuV1 capsid at pH 7.4 (green), 4.0 (teal), and 2.6 (tan).

The previously solved structure of BuV1 at pH 7.4 (PDB: 6BWX) was used as the initial model for all three data sets and docked into the reconstructed density map. Following manual and automated refinement, final models for all three data sets were produced. To determine differences between the BuV1 capsid at different pHs, a monomer was extracted from each 60-mer and overlayed using secondary structure matching to the pH 7.4 monomer (Figure 6A). Overall, the capsids superimposed well, with a RMSD of 0.75 Å between pH 7.4 and 4.0 and a RMSD of 0.36 Å between pH 7.4 and 2.6. However, there were two major differences between the capsid structures, localized at the N-terminus and in VR-IX (Figure 6B,C). At pH 2.6, the ordering of the N-terminus allowed for the assignment of 10 additional residues compared to either pH 7.4 or 4.0 structures (Figure 6B). Furthermore, minor rearrangements were observed between the N-termini of pH 7.4 and 4.0 structures. Between residues N530-L547, pH-dependent loop rearrangements in VR-IX were observed (Figure 6C). This loop is located at the 2-fold depression of the BuV1 capsid and is responsible for the gross morphological differences observed. The large structural rearrangements at the 2-fold symmetry axis showed significant deviations in the structure superimposition between pH 7.4 and 2.6. Interestingly, it appears that at pH 4.0, an intermediate structural state is observed, with G540-L547 being more similar to the equivalent residues at pH 7.4, while N530-G540 are more similar to the equivalent residues at pH 2.6. Additionally, minor structural rearrangements were present between pH 7.4 and 4.0 structures, localized at VR-I, VR-II, VR-III, VR-IV, and VR-VII (Figure 6A).

### 3.5. Rearrangements in the 5-Fold Pore Suggest a pH-Specific Role of Q157

**Figure 7:**
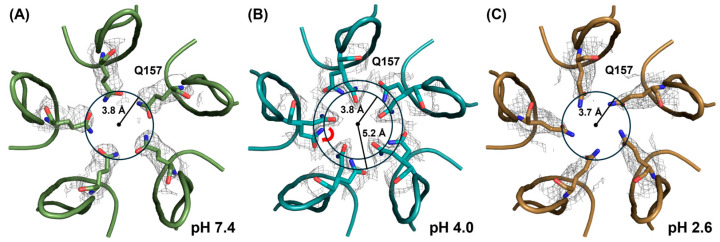
pH-dependent differences in the five-fold pore. Cartoon representation of the pentamers of BuV1 capsid around the 5-fold symmetry axis at (**A**) pH 7.4, (**B**) pH 4.0, and (**C**) pH 2.6. Q157 is shown in stick conformation with the electron density map overlayed at 1.5σ. The 5-fold pore is shown as a circle, defined by the C*δ* of Q157. The radius from the center is indicated in Å. The red arrow in B represents the 90° shift between the extended and compacted rotamers.

The 5-fold pores of the capsid provide a channel connecting the interior of the capsid to the exterior. Furthermore, this pore is hypothesized to be the site of VP1u externalization and the site of genome ejection [23,26,27]. An analysis of the 5-fold pore showed that Q157 exhibits a unique rotamer at different pH conditions (Figure 7). At pH 7.4 and 2.6, the rotamer is in an extended conformation, resulting in a pore with an approximate radius of 3.8 Å (Figure 7A,C). However, at pH 4.0, the presence of two rotamers, one in an extended conformation and the other in a more compacted conformation, results in a pore with an approximate radius of either 3.8 or 5.2 Å, respectively (Figure 7B). The extended rotamer has a Cα-Cβ torsion angle of −164°, while the compacted rotamer has a Cα-Cβ torsion angle of −70°, indicating a shift of approximately 90°. Since both rotamers are present, it is likely that the capsid exhibits both states, with perhaps one state being more favored under certain physiological conditions.

## 4. Discussion

There is very little current knowledge available on BuV1, especially at the molecular level, partially due to it being a relatively newly discovered virus. To add to this knowledge, we first sought to determine if a terminal glycan was responsible for cell binding. Subsequently, we attempted to locate the site of glycan attachment on the capsid surface. The glycan array, cell binding data, and structural characterization of BuV1 in complex with 3SLN and 6SLN revealed that only the terminal sialic acid moiety is responsible for binding to the capsid. This is similar to a previous report of serpentine adeno-associated virus (SAAV) complexed with 3SLN and 6SLN [56]. Furthermore, complex structures of AAV1, AAV5, AAV6, and MVMp with SIA have been determined [34,52,57]. However, it is interesting to note that the SIA binding site differs between viruses (Supplementary Figure S3A). While SIA binds both AAV1 and AAV5 near the 3-fold protrusion, for BuV1, SAAV, and MVMp, SIA binds near the 2-fold depression. The binding site for each parvovirus determined so far is unique, generally clustering around the 3-fold or 2-fold axes. However, an overlay of the SIA binding pocket from BuV1 with the structures of BuV2 and BuV3 at pH 7.4 demonstrates that the binding pocket is conserved between these viruses, suggesting that SIA is likely important for cell attachment for these related viruses (Supplementary Figure S3B). Additionally, many protoparvoviruses, such as CPV, FPV, PPV, H-1PV, and LuIII, as well as the two human protoparvoviruses, cutavirus and tusavirus, have been previously shown to bind to glycans with a terminal sialic acid, suggesting commonality between these infectious agents [38,58]. Future experiments will be needed to confirm whether BuV1 binds cells using SIA to mediate endocytosis in cells and in vivo. Furthermore, if SIA is shown to mediate endocytosis in cells, small molecules could be designed to the SIA binding pocket of BuV1 to act as anti-virals to prevent infection.

Structural and biophysical characterization of BuV1 showed that the capsid undergoes rearrangements in response to pHs it encounters during the infection process. We observed stabilization of the BuV1 capsid at pHs, which corresponds to acidification encountered during the endo–lysosomal pathway (Figure 4). Stabilization at pH 4.0 likely serves a dual purpose during viral infection and endo–lysosomal escape. As BuV1 transits through the host gastrointestinal system, including the stomach, where pH conditions range from pH 1.5 to 3.5, the virus may be vulnerable to the environment during this mode of transmission [53,54,55]. Thus, BuV1 may require a temporary rise of the pH through food and liquid uptake or inflammation to survive in the stomach before transitioning into the intestine, where the pH is more neutral [53,54,55]. Here, the BuV1 capsid is stable and could attach to intestinal cells, which present terminal sialic acid glycans. After endocytosis, stabilization of the BuV1 capsid at lower pH values would allow for the externalization of VP1u, allowing for endo–lysosomal escape. We could not obtain a measurable T_m_ of BuV1 at pH 2.6. However, prior work has shown that human bocavirus 1 (HBoV1), human bocavirus 2 (HBoV2), and AAV5 can be analyzed by differential scanning fluorimetry at pH 2.6, so it is possible that BuV1 is too unstable at low pH, perhaps due to the “broken” particles we observed preventing a reliable readout [59]. 

Structural characterization of capsids at pH 7.4, 4.0, and 2.6 revealed rearrangements that could explain the differences in thermal stability observed. For instance, the large rearrangement in VR-IX observed at pH 2.6 occurs at the 2-fold axis of symmetry, which suggests that this may destabilize the 2-fold interface. Indeed, in micrographs of the BuV1 capsid at pH 2.6, capsids were observed that appeared “broken” (Figure 5C, red arrow). The ordering of additional residues at the N-terminal was also observed at pH 2.6, similar to the HBoV1 and HBoV2 capsids [59]. Additionally, the smaller rearrangements observed at pH 4.0 in VR-I, VR-II, VR-III, VR-IV, and VR-VII may serve to stabilize the BuV1 capsid during conditions similar to those in a lysosome. Interestingly, the overlay of the structures at different pH values with the BuV1-6SLN structure showed that the SIA binding site was largely unchanged throughout (Supplementary Figure S4). Perhaps under physiological conditions, other factors ensure that BuV1 capsids release from their bound receptor to facilitate downstream steps in the viral infection pathway. 

**Figure 8:**
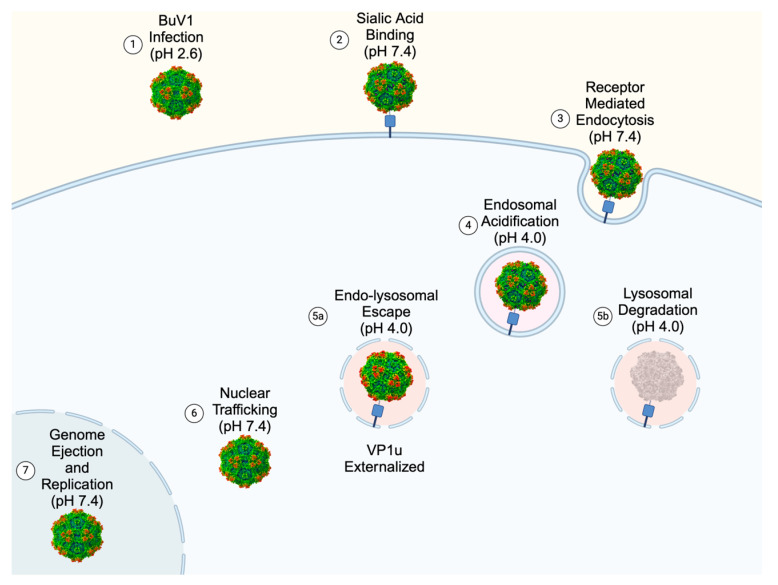
Structures of BuV1 yield insight into the viral lifecycle. Diagram showing the proposed viral lifecycle gleaned from the biophysical and structural characterization of BuV1 at different stages of the viral lifecycle. Reconstructed electron density maps are shown at 2σ as follows: Step 1: pH 2.6, Steps 2–4: 6SLN, Step 5a and 5b: pH 4.0, and Step 6–7: pH 7.4. VP1u externalized is postulated due to the alternative conformation of Q157 observed at pH 4.0, which leads to a 5-fold pore with a wider radius.

Finally, structural rearrangements of the 5-fold pore at various pHs provide a glimpse of a potential step in the viral lifecycle (Figure 8). At pH 2.6, when the virus is presumably in the stomach, Q157 adopts an extended conformation that restricts the radius of the 5-fold pore, preventing premature externalization of VP1u or the viral genome. The virions that survive the stomach then traffic to the intestine, where, at pH 7.4, Q157 also adopts an extended conformation, further preventing VP1u externalization or genome ejection. Once the virus binds SIA and undergoes receptor-mediated endocytosis, acidification during the endo–lysosomal pathway causes Q157 to shift conformations at pH 4.0, resulting in a larger pore radius, allowing for the externalization of VP1u and facilitating endo–lysosomal escape for some of the virions. Some virions likely do not escape and are degraded in the lysosome. However, virions that do escape subsequently traffic to the nucleus and undergo genome ejection, therefore starting the process of producing more virions. 

Here, detailed structural characterizations of BuV1 during several steps of the viral lifecycle are presented, thereby helping to better understand this pathogenic parvovirus. Moving forward, the information gained from this study could be applied to other pathogenic parvoviruses and potentially help inform therapeutic interventions for individuals suffering from BuV1-correlated gastroenteritis. 

## Figures and Tables

**Table 1:** Cryo-EM Data Collection and Refinement Statistics.

**Data Collection**				
Data set	BuV1 6SLN	BuV1 pH 7.4	BuV1 pH 4.0	BuV1 pH 2.6
PDB ID	9CUZ	9CV0	9CV9	9CWS
EMDB ID	EMD–45954	EMD–45955	EMD–45958	EMD–45973
Total number of micrographs	1115	1961	827	1496
Defocus range (μm)	0.1–3.6	0.1–4.0	0.1–2.8	0.1–2.9
Electron dose (e^-^/Å^2^)	75	75	75	75
Frames per micrograph	50	50	50	50
Pixel size (Å/pixel)	1.068	1.050	1.051	1.056
Number of particles used	95,075	55,466	55,060	121,222
Resolution (0.143 FSC)	2.16	2.84	3.20	2.73
**Model Refinement Statistics**				
Map correlation coefficient	0.88	0.85	0.77	0.86
RMSD bond lengths (Å)	0.011	0.011	0.010	0.010
RMSD bond angles (°)	1.316	1.287	0.863	0.965
All-atom clash score	14.03	17.28	9.95	14.02
**Model Validation**				
Ramachandran Favored (%)	94.95	94.58	89.91	93.94
Ramachandran Allowed (%)	5.05	5.42	10.09	5.69
Ramachandran Outliers (%)	0	0	0	0
Rotamer Outliers (%)	0.42	0	0	0.2
C-β deviations (%)	0	0	0	0

## Data Availability

The final reconstructed density maps and models were deposited in the Protein Data Bank (PDB) and Electron Microscopy Data Bank (EMDB) as follows: 6SLN—PDB: 9CUZ, EMDB: EMD-45954; pH 7.4—PDB: 9CV0, EMDB: EMD-45955; pH 4.0—PDB: 9CV9, EMDB: EMD-45958; and pH 2.6—PDB: 9CWS, EMDB: EMD-45973.

## Supplementary Materials

The following supporting information can be downloaded at: https://www.mdpi.com/article/10.3390/v16081258/s1; Figure S1 Extended Glycan Array Hits; Figure S2 BuV1 electron density maps and atomic models; Figure S3 The sialic acid binding site is likely conserved between bufavirus serotypes but not between distinct parvoviruses; Figure S4 The sialic acid binding site is insensitive to pH.
