# Supplementary material for: Structural Characterization of Human Bufavirus 1: Receptor Binding and Endosomal pH-Induced Changes
Source: Viruses. 2024 Aug 6;16(8):1258. doi: 10.3390/v16081258 (PMC11360561; doi:10.3390/v16081258)
Supplement: Supplementary file 1 [file viruses-16-01258-s001.zip › viruses-3110490-supplementary.pdf]

Article

Structural Characterization of Human Bufavirus 1: Receptor Binding and Endosomal pH-Induced Changes

Mitchell Gulkis [1], Mengxiao Luo [1], Paul Chipman [1], Mario Mietzsch [1], Maria Söderlund-Venermo [2], Antonette Bennett [1,*] and Robert McKenna [1,*]

[1] Department of Biochemistry and Molecular Biology, University of Florida, Gainesville, FL 32611, USA; mitchell.gulkis@ufl.edu (M.G.); mxluo@connect.hku.hk (M.L.); pchipman@ufl.edu (P.C.); mario.mietzsch@ufl.edu (M.M.)
[2] Department of Virology, University of Helsinki, P.O. Box 21 (Haartmaninkatu 3), FIN-00014 Helsinki, Finland; maria.soderlund-venermo@helsinki.fi
* Correspondence: dendena@ufl.edu (A.B.); rmckenna@ufl.edu (R.M.)

Supplemental Figures 1–4

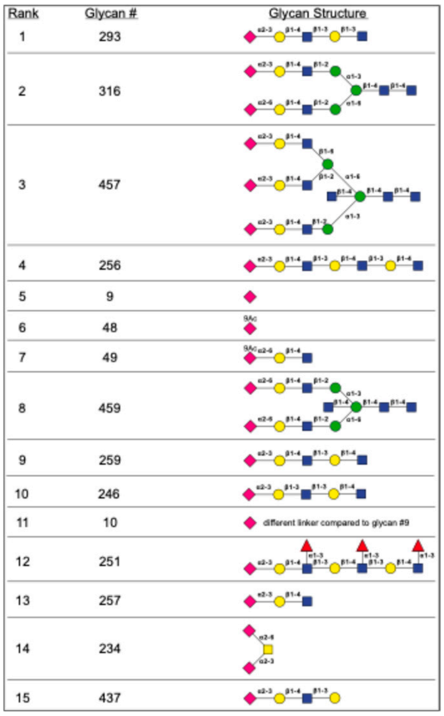

Rank	Glycan #	Glycan Structure
1	293	
2	316	
3	457	
4	256	
5	9	
6	48	
7	49	
8	459	
9	259	
10	246	
11	10	different linker compared to glycan #9
12	251	
13	257	
14	234	
15	437	

Supplementary Figure 1. Extended Glycan Array Hits. Table showing the symbols of the glycan structure of the top five glycans with the highest signal. Pink diamonds represent N-acetylneuraminic acid, yellow circles represent galactose, blue squares represent N-acetylglucosamine, and green circles represent mannose. Glycosidic linkages are indicated between sugars.

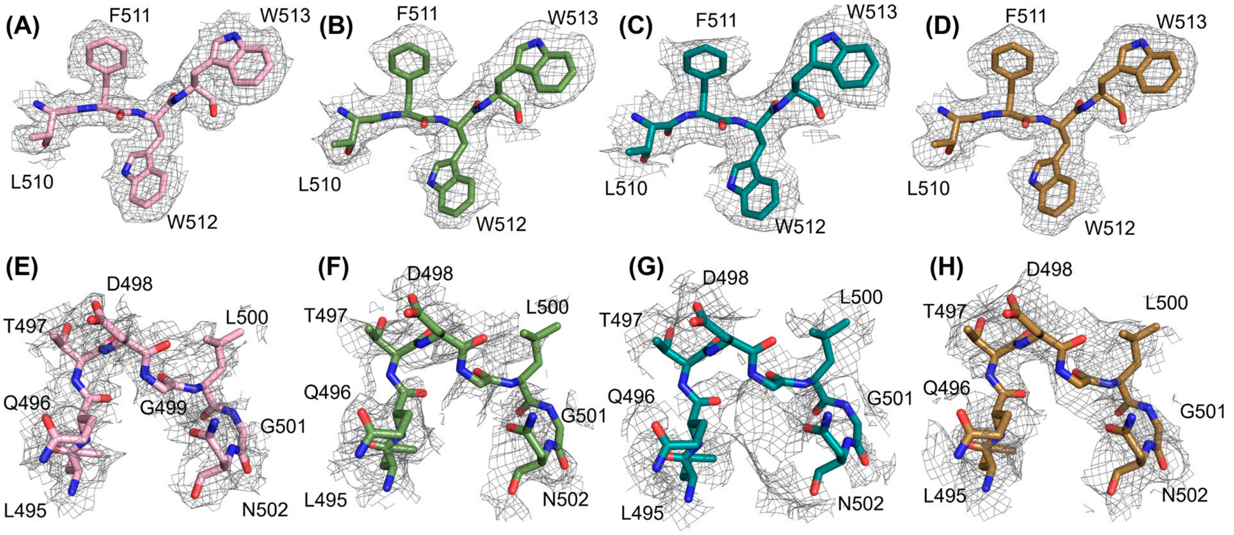

Supplementary Figure 2. BuV1 electron density maps and atomic models. (A-D) Electron density is shown around L510-W513 contoured at 1σ for BuV1-6SLN (A), pH 7.4 (B), pH 4.0 (C), and pH 2.6 (D). The atomic model fits the density well. (E-H) Electron density is shown around L495-N502, located in the HI loop, at 1σ for BuV1-6SLN (E), pH 7.4 (F), pH 4.0 (G), and pH 2.6 (H). The poor electron density precluded building an atomic model with high confidence.

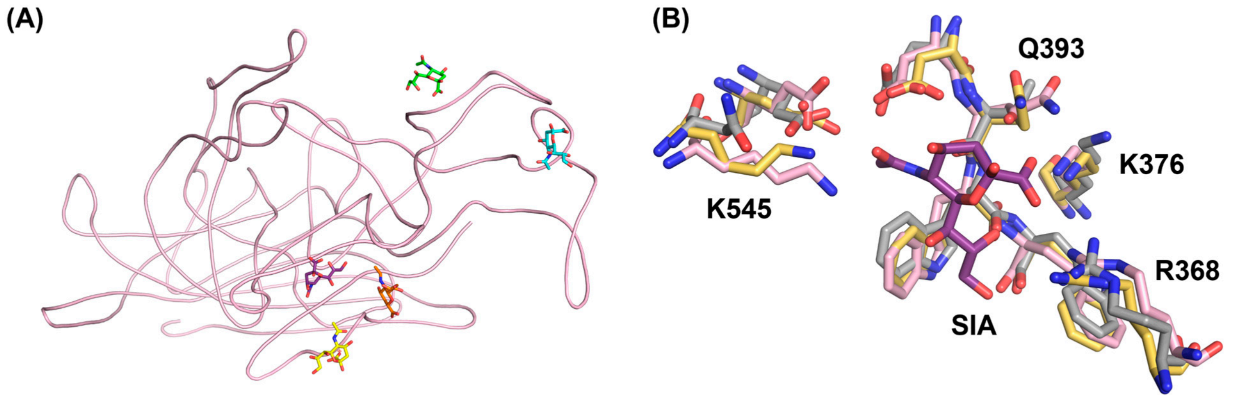

Supplementary Figure 3. The sialic acid binding site is likely conserved between bufavirus serotypes but not between distinct parvoviruses. (A) The binding site of sialic acid is shown for BuV1 (purple), AAV1 (cyan), AAV5 (green), SAAV (yellow), and MVMp (orange). (B) The sialic acid binding site is shown for BuV1 (pink), BuV2 (grey), and BuV3 (yellow). Residues which interact with sialic acid in the BUV1 structure are annotated.

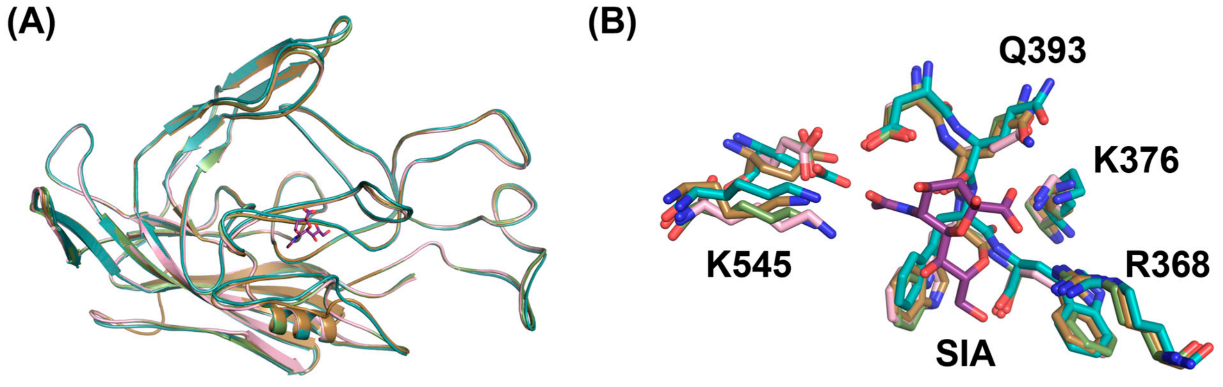

Supplementary Figure 4. The sialic acid binding site is insensitive to pH. (A) Overlay of BuV1-6SLN (pink), pH 7.4 (green), pH 4.0 (teal), and pH 2.6 (tan). Sialic acid is shown in stick form in purple. (B) Zoomed view of the sialic acid binding site. Interacting residues colored as in A and are annotated. No major positional shifts are observed at lower pH values.
